# Regulation of glutamate transporter trafficking by Nedd4-2 in a Parkinson's disease model

**DOI:** 10.1038/cddis.2016.454

**Published:** 2017-02-02

**Authors:** Yunlong Zhang, Xiaoliang He, Xingjun Meng, Xiaojuan Wu, Huichun Tong, Xiuping Zhang, Shaogang Qu

**Affiliations:** 1 Department of Neurology, The First People's Hospital of Shunde Affiliated to Southern Medical University, Foshan, Guangdong 528300, China; 2 Department of Immunology, School of Basic Medical Sciences, Southern Medical University, Guangzhou, Guangdong 510515, China; 3 Teaching Center of Experimental Medicine, School of Basic Medical Sciences, Southern Medical University, Guangzhou, Guangdong 510515, China

## Abstract

Glutamate transporters play a key role in glutamate clearance and protect the central nervous system from glutamate excitotoxicity. Dysfunctional glutamate transporters contribute to the pathogenesis of Parkinson's disease (PD); however, the mechanisms that underlie the regulation of glutamate transporters in PD are still not well characterized. Here we report that Nedd4-2 mediates the ubiquitination of glutamate transporters in 1-methyl-4- phenylpyridinium (MPP^+^)-treated astrocytes and in the midbrain of 1-methyl-4-phenyl-1,2,3,6- tetrahydropyridine (MPTP)-constructed PD model mice. Nedd4-2-mediated ubiquitination induces abnormal glutamate transporter trafficking between the membrane and cytoplasm and consequently decreases the expression and function of glutamate transporters in the membrane. Conversely, Nedd4-2 knockdown decreases glutamate transporter ubiquitination, promotes glutamate uptake and increases glutamate transporter expression *in vitro* and *in vivo*. We report for the first time that Nedd4-2 knockdown ameliorates movement disorders in PD mice and increases tyrosine hydroxylase expression in the midbrain and striatum of PD mice; Nedd4-2 knockdown also attenuates astrogliosis and reactive microgliosis in the MPTP model that may be associated with glutamate excitotoxicity. Furthermore, the SGK/PKC pathway is regulated downstream of Nedd4-2 in MPTP-treated mice. These findings indicate that Nedd4-2 may serve as a potential therapeutic target for the treatment of PD.

Glutamate excitotoxicity contributes to the pathogenesis of Parkinson's disease (PD), and sporadic PD patients show decreased platelet glutamate uptake, which correlates with disease severity.^1, 2^ Excitatory amino acid transporters (EAATs) play a predominant role in clearing the excessive glutamate in the synaptic cleft. Five mammalian EAATs have been characterized: glutamate/aspartate transporter (GLAST, also called EAAT1), glutamate transporter-1 (GLT-1, also called EAAT2), excitatory amino acid carrier-1 (EAAC1, also called EAAT3), EAAT4 and EAAT5.^3, 4, 5, 6, 7, 8^ Among these five isoforms, GLT-1 is responsible for taking up nearly 90% of the glutamate in astrocytes. Decreased glutamate transporters expression and glutamate uptake have been observed in PD animal models generated using 6-hydroxydopamine, 1-methyl-4-phenyl-1,2,3,6- tetrahydropyridine (MPTP) and 1-methyl-4- phenylpyridinium (MPP^+^).^9, 10, 11, 12^ In addition, single unilateral injection of EAATs substrate inhibitor l-trans-pyrrolidine-2,4-dicarboxylate induces DA neurons death, with motor deficits caused by 50% loss of neurons.^13^ The latter model mimics several PD features and indicates that dysfunction of EAATs participates in PD.^13^ Medications or molecules that target glutamate transporters show benefits in neurodegenerative disease, such as ceftriaxone, LDN/OSU-0212320 and iptakalim.^14, 15, 16^ Furthermore, upregulation of glutamate transporters also block the abnormal accumulation of *α*-synuclein, *β*-amyloid and tau.^17, 18, 19^

Glutamate transporters also play an important role in maintaining homeostasis for excitatory glutamate transmission within synapses formed by neurons and astrocytes. GLT-1 ameliorates motor disorders induced by glutamate excitotoxicity, and blockade of GLT-1 in the central amygdala is associated with symptoms of depression and anxiety.^20, 21^ Previously we showed that ceftriaxone can improve MPP^+^-induced abnormal distribution of GLT-1 in astrocytes.^22^ In addition, ceftriaxone promotes the membrane expression of GLT-1 in MPP^+^-treated astrocytes and attenuates the apoptosis of astrocytes via suppression of the NF-*κ*B/JNK/c-Jun signal pathway.^22^ These results suggest that glutamate transporters could provide a target in treating PD. However, the mechanism of abnormal glutamate transporter trafficking in the membrane and cytoplasm in PD is still unclear.

Ubiquitination is a crucial process in modulating the distribution of intracellular proteins. Generally, ubiquitin is coupled to lysine residues on target proteins by a cascade of reactions carried out sequentially by the ubiquitin-activating enzyme (E1), ubiquitin-conjugating enzyme (E2) and ubiquitin ligase enzyme (E3). E3 ligases can be sub-divided into three types: really interesting new gene (RING)-type (including U-box ligases), homologous with E6 associated protein C terminus (HECT)-type and RING-HECT hybrids.^23, 24^ The E3 ligases recognize the target proteins and thus impart specificity to the ubiquitination process. Consequently, E3 ligases play important roles in the pathogenesis of PD. For example, Parkin, an E3 ubiquitin ligase that complexes with PINK1 and DJ-1 to promote degradation of misfolded proteins, is associated with familial PD.^25, 26^

Nedd4-2 (neuronal precursor cell expressed developmentally down-regulated 4-2) is a member of the HECT family. Nedd4-2 can mediate the ubiquitination of epithelial sodium channel (ENaC), voltage-gated sodium channels (Na_v_s) and also the glutamate transporters.^27, 28, 29, 30, 31^ however, it is unclear whether Nedd4-2-mediated ubiquitination of glutamate transporters plays a role in PD and whether Nedd4-2 may potentially serve as a therapeutic target in PD.

In this study, we provide evidence that Nedd4-2 mediates the ubiquitination of glutamate transporters in *in vitro* and *in vivo* of PD models. Nedd4-2 knockdown decreases the ubiquitination of glutamate transporters, promotes glutamate uptake, and increases the expression of glutamate transporters *in vitro* and *in vivo*. Notably, we demonstrate for the first time that Nedd4-2 knockdown ameliorates the movement disorder in PD mice and increases tyrosine hydroxylase (TH) expression in PD mice. Hence, we propose that Nedd4-2 represents a potential therapeutic target for the treatment of PD.

## Results

### Nedd4-2 mediates the ubiquitination of glutamate transporter GLT-1 in MPP^+^-treated astrocytes

**Figure 1:**
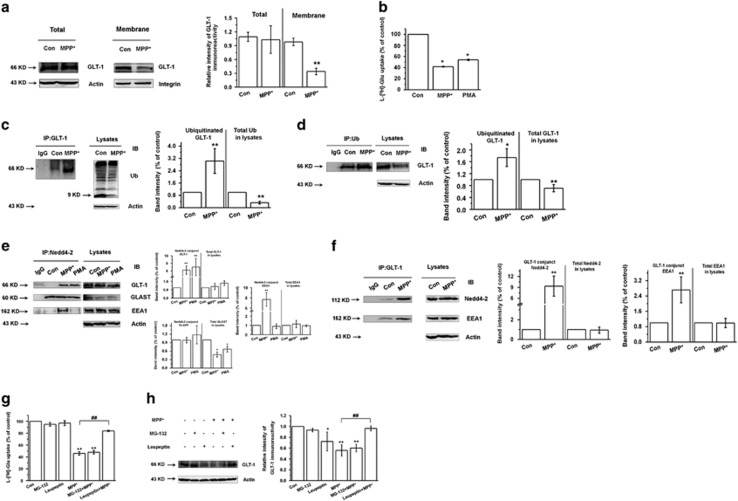
Nedd4-2 mediates the ubiquitination of glutamate transporter GLT-1 in MPP^+^-treated astrocytes. (**a**) Western blotting analysis showing that GLT-1 expression is decreased on MPP^+^ treatment of astrocytes for 24 h in the membrane. (**b**) l-[^3^H]-Glutamic acid uptake assay showing that 1 mM MPP^+^ and 2 *μ*M PMA treatment for 24 h decreases the glutamate uptake in astrocytes. (**c** and **d**) Co-IP assays showing that GLT-1 is ubiquitinated after 24 h MPP^+^ treatment of astrocytes. The capture antibody in **c** was anti-GLT-1; and in (**d**) was anti-Ub. IgG was tested as a negative control. (**e** and **f**) Co-IP assay showing the interaction between Nedd4-2 and GLT-1 in MPP^+^ and PMA-treated astrocytes. The capture antibody in (**e**) was anti-Nedd4-2 and MPP^+^ treatment time was 24 h; the capture antibody in (**f**) was anti-GLT-1 and MPP^+^ treatment time was 24 h. IgG was tested as a negative control. (**g**) l-[^3^H]-Glutamic acid uptake assay showing that lysosome inhibitor (Leupeptin) while not proteasome inhibitor (MG-132) increased glutamate uptake in MPP^+^-treated astrocytes for 24 h. (**h**) Western blotting analysis showing that Leupeptin while not MG-132 increased GLT-1 expression in MPP^+^-treated astrocytes for 24 h. Results are expressed as the mean±S.E. of at least three experiments. Student's *t*-test for Figures 1a, c and d, and One-way ANOVA for Figures 1b, e, f, g and h. *Represents a significant difference between other group and control group. **P<0.01, *P<0.05. ^#^Represents a significant difference between MPP^+^ group and Leupeptin+MPP^+^ group. ^#^*P*<0.05

To characterize the effect of MPP^+^ on glutamate transport and the potential role of ubiquitination, we first examined GLT-1 expression in MPP^+^-treated astrocytes for 24 and 48 h. GLT-1 expression and glutamate uptake were obviously decreased after 24 and 48 h of MPP^+^ treatment (Figures 1a and b and Supplementary Figure 1a–c), which is consistent with our previous results.^22^ As PKC has been reported to promote Nedd4-2-mediated ubiquitination of glutamate transporters.^27, 28, 29^ Here we also show that phorbol-12-myristate- 13- acetate (PMA, PKC activator) decreased glutamate uptake after 24 and 48 h (Figure 1b and Supplementary Figure 1d), although the opposite effect was observed after 20 min PMA treatment (Supplementary Figure 1e).

To determine whether the ubiquitination of GLT-1 is induced by MPP^+^, we performed co-immunoprecipitation (co-IP) assays. We suggest that GLT-1 is ubiquitinated in MPP^+^-treated astrocytes (Figures 1c and d), but ubiquitin did not pull down Nedd4-2, GLAST, or early endosomal autoantigen 1 (EEA1), an endosomal protein which is reported to be colocalized with ubiquitinated GLT-1 (data not shown).^32, 33^ Nedd4-2 is thought to mediate GLT-1 ubiquitination in a PKC-dependent manner.^27, 29, 33, 34, 35, 36^ To determine whether Nedd4-2 mediates GLT-1 ubiquitination in MPP^+^-treated astrocytes, we examined Nedd4-2 expression and its interaction with GLT-1. The expression of phosphorylated and total Nedd4-2 was not significantly different in control versus MPP^+^-treated astrocytes (Supplementary Figure 1f). However, MPP^+^ treatment at 24 and 48 h promoted the interaction between Nedd4-2 and GLT-1 (Figure 1e and Supplementary Figure 1g). PMA also induced an interaction between Nedd4-2 and GLT-1 at 24 h, but not at 48 h of treatment (Figure 1e and Supplementary Figure 1g). In addition, MPP^+^ enhanced the interaction between Nedd4-2 and EEA1 at both 24 and 48 h, but the interaction between Nedd4-2 and GLAST was not affected by MPP^+^ treatment (Figure 1e and Supplementary Figure 1g). Using GLT-1 as capture antibody, we verified the interaction of GLT-1 with Nedd4-2 and EEA1 in MPP^+^-treated astrocytes (Fig. 1f; *P*<0.01). These findings suggest that upon ubiquitination by Nedd4-2, GLT-1 is sorted to the early endosome. As GLT-1 is reported to be targeted for lysosomal degradation with prolonged activation of PKC and PMA-induced GLT-1 redistribution could be reversed by inhibiting lysosomal degradation,^37^ in this study we also draw the similar conclusion. We found that lysosome inhibitor Leupeptin increased glutamate uptake and GLT-1 expression in MPP^+^-treated astrocytes, while proteasome inhibitor MG-132 showed no obvious effects (Supplementary Figure 2a and Figures 1g and h). These results suggest that MPP^+^-decreased GLT-1 can be rescued by inhibiting lysosomal degradation and MPP^+^ may induce GLT-1 for ubiquitination, endocytosis to EEA1 and degradation in the lysosome but not proteasome in astrocytes.

Because GLT-1 does not bear the binding targets of Nedd4-2, we considered the possibility that an intermediate protein may mediate their interactions. Caveolin-1 mediates the endocytosis of several membrane proteins, including EAAC1.^38, 39, 40, 41^ Furthermore, caveolin-1 can decrease ENaC expression via a mechanism that involves the promotion of Nedd4-2-dependent internalization of ENaC.^42^ These results suggest that caveolin-1 can mediate the internalization of substrates of Nedd4-2. Therefore, we also performed reciprocal co-IP assays to assess the interaction of caveolin-1 with Nedd4-2 and GLT-1. Our results demonstrate that both Nedd4-2 and GLT-1 interacted reciprocally with caveolin-1 (Supplementary Figures 2b and c). Thus, our findings raise the possibility that Nedd4-2 may mediate the ubiquitination of GLT-1 in MPP^+^-treated astrocytes via interaction with caveolin-1.

### Nedd4-2 knockdown decreases the ubiquitination of GLT-1, promotes GLT-1 expression and increases glutamate uptake in MPP^+^-treated astrocytes

**Figure 2:**
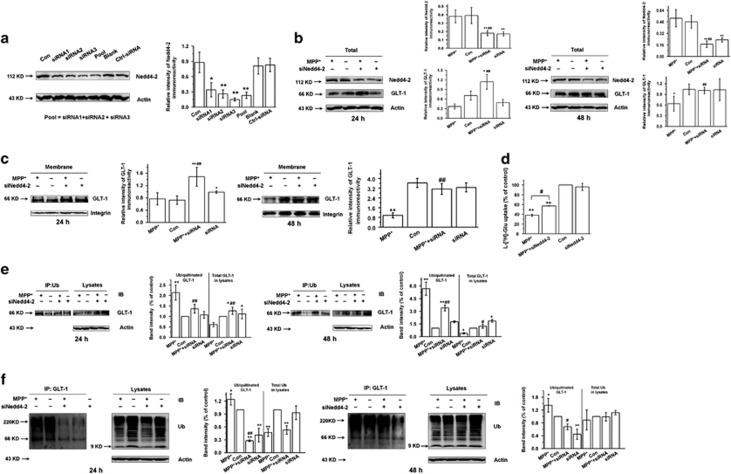
Nedd4-2 knockdown decreases ubiquitinated GLT-1 expression and increases GLT-1 expression and glutamate uptake in MPP^+^-treated astrocytes. (**a**) Western blotting showing the Nedd4-2 expression in astrocytes at 72 h after transfection with three different Nedd4-2 siRNAs, a mixture of the three siRNA, PBS or negative control siRNA. (**b**) Western blotting showing that Nedd4-2 knockdown increases total GLT-1 expression levels after MPP^+^ treatment of astrocytes for 24 h or 48 h. (**c**) Western blotting showing that Nedd4-2 knockdown increases membrane GLT-1 levels after MPP^+^ treatment of astrocytes for 24 h or 48 h. (**d**) l-[^3^H]-Glutamic acid uptake assay showing the effects of Nedd4-2 knockdown on glutamate uptake after MPP^+^ treatment of astrocytes for 24 h. Nedd4-2 knockdown promoted glutamate uptake after 24 h MPP^+^ treatment. (**e**,**f**) Co-IP assay showing that Nedd4-2 knockdown decreases the ubiquitination of GLT-1 in astrocytes after 24 h or 48 h MPP^+^ treatment. The capture antibody in (**e**) was anti-Ub; the capture antibody in (**f**) was anti-GLT-1. Results are expressed as the mean±S.E. of at least three experiments. One-way ANOVA. **P<0.01, *P<0.05, ^##^*P*<0.01, ^#^*P*<0.05, *represents a significant difference between other group and control group; while ^#^represents a significant difference between MPP^+^+siRNA group and MPP^+^-treated astrocytes

We further transfected three different synthetic siRNAs (Nedd4-2 siRNA-1, 2 and 3) into astrocytes to specifically knockdown Nedd4-2 expression. The knockdown efficiency of Nedd4-2 siRNA-3 in astrocytes was 70–80% (Figure 2a and Supplementary Figure 2d*; P*<0.01), and therefore, we used siRNA-3 for subsequent assays. We demonstrate that Nedd4-2 knockdown increased total (Figure 2b) and membrane (Figure 2c) GLT-1 levels after 24 h and 48 h of MPP^+^ treatment in astrocytes (*P*<0.01). Furthermore, at 24 h Nedd4-2 knockdown increased glutamate uptake (Figure 2d; *P*<0.05), which corresponds to the elevated GLT-1 levels (Figures 2b and c). Nedd4-2 knockdown also decreased the ubiquitination of GLT-1 in astrocytes at both 24 and 48 h of MPP^+^-treatment (Figures 2e and f; *P*<0.01 and *P*<0.05, respectively). Therefore, these results provide direct evidence that Nedd4-2 mediates the ubiquitination of GLT-1, which may explain the decreased GLT-1 levels and glutamate uptake in MPP^+^-treated astrocytes.

### The expression and function of glutamate transporters is decreased in MPTP-treated mice

**Figure 3:**
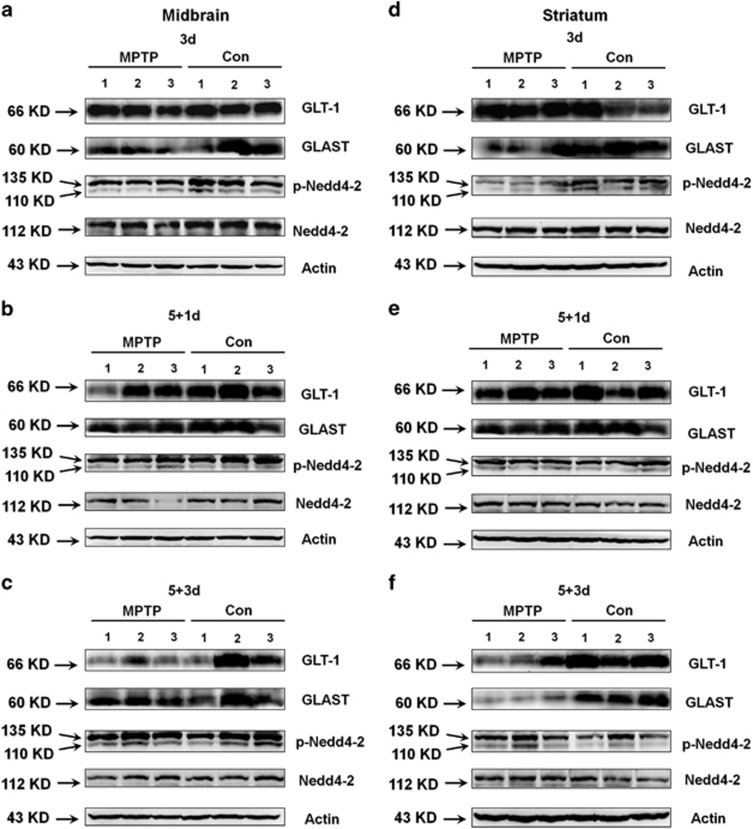
The protein expression of glutamate transporters is decreased in the midbrain and striatum in MPTP-treated mice. C57BL/6 mice received one intraperitoneal injection of MPTP-HCl or saline daily for 3 days and then were killed (3d) or received one intraperitoneal injection of MPTP-HCl or saline daily for 5 days and were killed after either 1 (5+1d) or 3 day (5+3d). The midbrain and striatum tissues were prepared for analysis by western blotting. (**a**–**c**) GLAST expression was decreased in the 3d group, and GLT-1 expression was decreased in the 5+3d group in the midbrain of MPTP-treated mice. *n*=3 per group. (**d**–**f**) GLT-1 expression was decreased in the 5+3d group, and GLAST expression was decreased in the 3d and 5+3d groups in the striatum of MPTP-treated mice. p-Nedd4-2/Nedd4-2 expression was decreased in the 3d group in the striatum of MPTP-treated mice. *n*=3 per group. Quantification of band intensities was provided in Supplementary Figures 4 and 5

**Figure 4:**
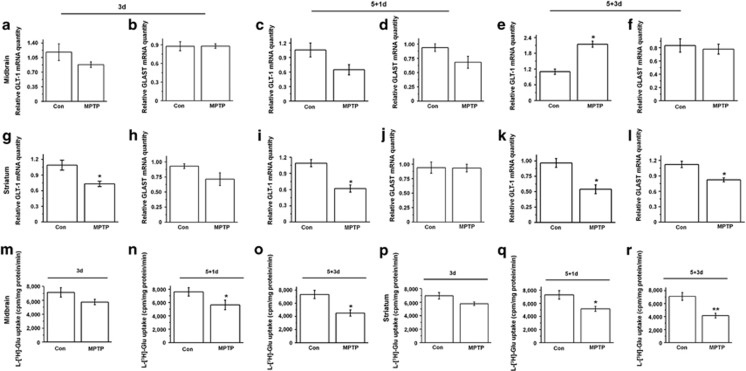
The mRNA expression of glutamate transporters and glutamate uptake in the synaptosome are decreased in MPTP-treated mice. Midbrain and striatum tissue were prepared from 3d, 5+1d and 5+3d groups of mice. (**a**–**f**) qPCR analysis showing that GLT-1 mRNA expression is increased in the 3d group (**e**). (**g**–**l**) qPCR analysis showing that GLT-1 mRNA expression was decreased in the 3d, 5+1d and 5+3d groups in the striatum (**g**, **i** and **k**). GLAST mRNA expression was decreased in the 5+3d group in the striatum (**l**). (**m**–**r**) The glutamate uptake in the synaptosomes was decreased in the 5+1d and 5+3d groups in the midbrain and striatum (**n**, **o**, **q** and **r**). Results are expressed as the mean±S.E. *n*=6 per group. Student's *t*-test. **P<0.01, *P<0.05

To determine whether GLT-1 exhibits a similar pattern of expression and regulation *in vivo*, we examined MPTP-treated mice. On MPTP treatment, mice displayed obvious movement deficits (Supplementary Figures 3a and b) and had reduced TH expression in the SN and striatum (Supplementary Figures 3c and d), which verifies that the PD model was generated successfully. To explore the dynamic changes of glutamate transporters in different time course in PD, we assessed glutamate transporters expression at protein and mRNA levels after 3 days MPTP injection (3d), the day after 5 daily MPTP injections (5+1d) and 3 days after 5 daily MPTP injections (5+3d) (Figures 3 and 4). We showed that MPTP treatment reduced GLT-1 expression in the 5+3d group in the midbrain (Figure 3c and Supplementary Figure 4g) and striatum (Figure 3f and Supplementary Figure 5g; *P*<0.05). MPTP treatment also reduced GLAST expression in the midbrain in the 3d group (Figure 3a and Supplementary Figure 4b) and in the striatum in the 3d and 5+3d groups (Figures 3d and f, Supplementary Figures 5b and h) (*P*<0.05); and decreased Nedd4-2 phosphorylation in the 3d group (Figure 3d and Supplementary Fig. 5c; *P*<0.05).

We also assessed mRNA expression of glutamate transporters in MPTP model (Figures 4a–l). Thereinto GLT-1 mRNA expression was statistically decreased in the striatum in the 3d, 5+1d and 5+3d MPTP-treated groups (Figures 4g, i and k; *P*<0.05). GLAST mRNA expression was statistically decreased in the striatum in the 5+3d MPTP-treated group (panel l; *P*<0.05). Moreover, Nedd4-2 mRNA expression was decreased in the striatum in the 5+3d MPTP-treated group (Supplementary Figure 6f). These findings confirm the general trend of MPTP in reducing GLT-1 and GLAST levels and suggest that the regulation of these glutamate transporters may occur both at the mRNA and protein levels.

The glutamate uptake in the synaptosome was decreased somewhat in all MPTP treatment groups, and the differences reached statistical significance in the midbrain and striatum in the 5+1d and 5+3d groups (panels n, o, q and r; *P*<0.05 and *P*<0.01). Furthermore, GLT-1 and GLAST mRNA levels in the cerebral cortex were statistically decreased in the 5+3d group (Supplementary Figures 7a–i; *P*<0.05), and a corresponding decrease in glutamate uptake in the cerebral cortex was observed for the 5+3d group (Supplementary Figures 7j–l; *P*<0.05). These results indicate that MPTP administration decreases expression and function of glutamate transporters.

### Nedd4-2 knockdown reverses the decreased glutamate transporter expression and function in PD model

**Figure 5:**
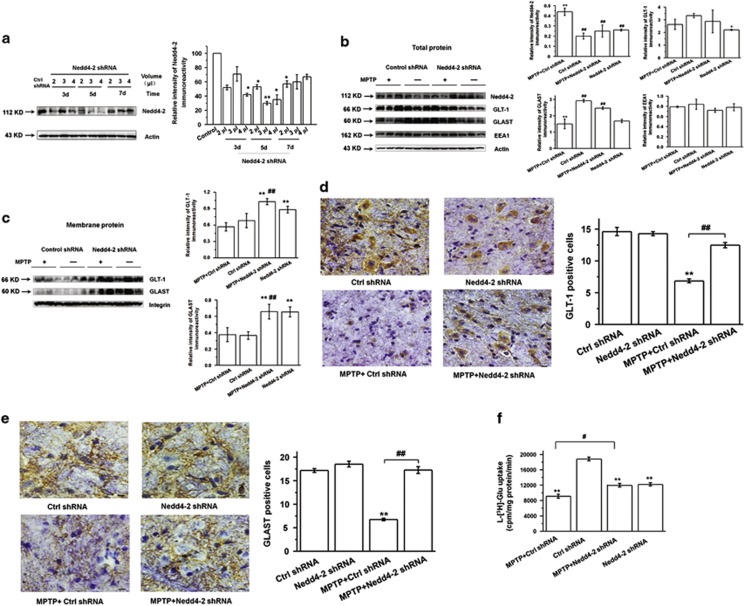
Nedd4-2 knockdown in MPTP-treated mice increases the expression and function of glutamate transporters. (**a**) Western blotting showing efficient lentiviral knockdown of Nedd4-2 in the midbrain of mice. 2.0, 3.0 or 4.0 *μ*l of virus were stereotaxically injected into the right side of the SN, and 3, 5 or 7 days after injection, the mice were killed for dissection of the midbrain. Nedd4-2 levels were measured by western blotting. Based on the results of this experiment, 3.0 *μ*l virus was injected into the SN for 5 days in subsequent experiments. (**b** and **c**) Western blotting showing that Nedd4-2 knockdown increases GLAST total protein expression (**b**) and GLT-1 and GLAST membrane protein expression (**c**). (**d** and **e**) Immunohistochemistry staining showing that Nedd4-2 knockdown increases GLT-1 and GLAST expression in the SN of MPTP-treated mice. Scale bar: 40 *μ*m. (**f**) Nedd4-2 knockdown increased glutamate uptake in the synaptosome in the midbrain of MPTP-treated mice. Results are expressed as the mean±S.E. One-way ANOVA. *n*=3 per group in (**a**–**c**). *n*=6 per group in (**d**–**f**). **P<0.01, *P<0.05, ^##^*P*<0.01, ^#^*P*<0.05. *Represents a significant difference between other group and ctrl shRNA group; while ^#^represents the significant difference between the indicated group and MPTP+ctrl shRNA group

To verify the role of Nedd4-2 in regulating glutamate transporters *in vivo*, we used lentivirus-mediated knockdown to reduce Nedd4-2 expression in PD mouse model. Nedd4-2 knockdown and control shRNA lentivirus were stereotaxically injected into the right side of the SN. We provide the detail vector map (Supplementary Figure 8a), and images of Nedd4-2 shRNA co-labeling with GFAP confirms that the lentivirus vector targets astrocytes in the SN (Supplementary Figure 8b). On the basis of our western blotting results (Figure 5a), in subsequent studies we selected to inject 3.0 *μ*l of virus into the SN and to begin the intaperitoneal injections of MPTP after 5 days following virus injection.

To determine whether Nedd4-2 knockdown modulates glutamate transporters in PD model, we performed western blotting and immunohistochemistry. Our results confirm that Nedd4-2 knockdown in MPTP-treated mice increased GLT-1 expression at the membrane protein level (Figure 5c; *P*<0.01). Nedd4-2 knockdown in MPTP-treated mice also increased GLAST expression at the total and membrane protein levels (Figures 5b and c; *P*<0.01). Increase of GLT-1 and GLAST levels in MPTP-treated mice upon treatment with Nedd4-2 shRNA was verified by immunostaining (Figures 5d and e and Supplementary Figures 8c and d; *P*<0.01). Moreover, Nedd4-2 knockdown increased the glutamate uptake in the synaptosome in the midbrain of MPTP-treated mice (Figure 5f; *P*<0.05). These findings suggest that Nedd4-2 knockdown can modulate glutamate transporters *in vivo*.

### Nedd4-2 interacts with GLT-1 and GLAST in MPTP-treated mice and mediates their ubiquitination

**Figure 6:**
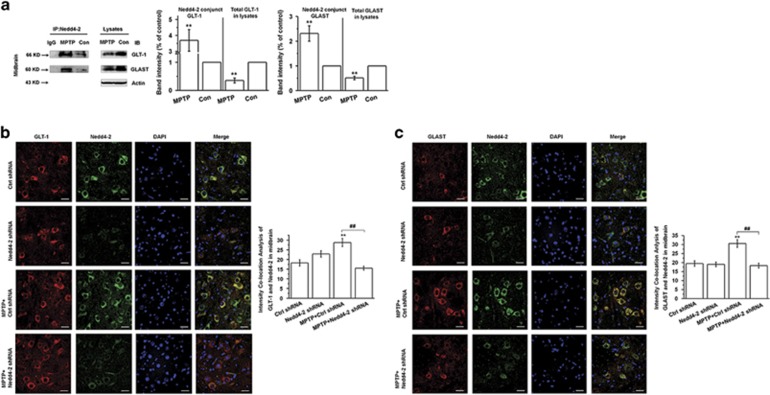
Nedd4-2 interacts with glutamate transporters and modulates their ubiquitination in the midbrain of MPTP-treated mice. C57BL/6 mice received one intraperitoneal injection of MPTP-HCl or saline daily for 5 days and were killed at day 3 after the last MPTP injection. (**a**) Immunoprecipitation assay showing that Nedd4-2 interacts with glutamate transporters in the midbrain of MPTP-treated mice. (**b** and **c**) Immunofluorescent staining showing that Nedd4-2 knockdown decreases the interaction between Nedd4-2 and glutamate transporters in SN in MPTP-treated mice. Results are expressed as the mean±S.E.. Student's *t*-test for Figure a. One-way ANOVA for **b** and **c**. *n*=6 per group. **P<0.01, ^##^*P*<0.01. *Represents a significant difference between other group and ctrl shRNA group; while ^#^represents the significant difference between MPTP+ctrl shRNA and MPTP+Nedd4-2 shRNA group. Scale bar: 30 *μ*m

Given our findings that GLT-1 is ubiquitinated and interacts with Nedd4-2 *in vitro*, we postulated that Nedd4-2 may also interact with glutamate transporters *in vivo* to mediate their ubiquitination and trafficking. Here we demonstrate that Nedd4-2 can interact with both GLT-1 and GLAST in the mouse midbrain, and that both interactions are enhanced after MPTP administration (Figure 6a; *P*<0.01). We provide the glutamate transporters localization on Nedd4-2 knockdown in SN in Figures 6b and c (*P*<0.01). These results suggest that Nedd4-2 mediates the ubiquitination of both GLT-1 and GLAST in the midbrain in MPTP-treated mice, and Nedd4-2 maybe a potential target in regulating glutamate transporters in PD.

### Nedd4-2 knockdown improves the behavior deficits and TH expression in PD model

**Figure 7:**
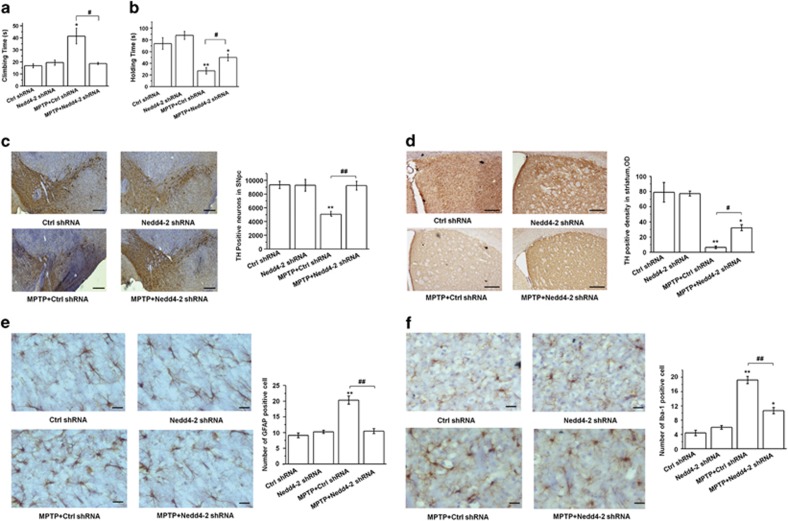
Nedd4-2 knockdown improves the motor disorder and TH expression in MPTP-treated mice. (**a** and **b**) Behavioral tests. Nedd4-2 knockdown prolonged the holding time and shortened the climbing time of MPTP-treated mice. (**c**) Immunohistochemistry (IHC) staining showing that Nedd4-2 knockdown increases TH expression in the SN in MPTP-treated mice. (**d**) IHC staining showing that Nedd4-2 knockdown increases TH expression in the striatum in MPTP-treated mice. (**e** and **f**) IHC staining showing that Nedd4-2 knockdown attenuates astrogliosis and reactive microgliosis in the SN in MPTP-treated mice. Results are expressed as the mean±S.E. One-way ANOVA. *n*=12 per group in (**a** and **b**); *n*=6 per group in (**c**–**f**). **P<0.01, *P<0.05, ^##^*P*<0.01, ^#^*P*<0.05. *Represents a significant difference between other group and ctrl shRNA group; while ^#^represents the significant difference between MPTP+ctrl shRNA and MPTP+Nedd4-2 shRNA group. Scale bar: 100 *μ*m in (**c**), 50 *μ*m in (**d**), and 30 *μ*m in (**e**) and (**f**)

To assess the functional outcome of Nedd-2 knockdown in the PD mouse model, we performed behavioral tests. Nedd4-2 shRNA prolonged the holding time and shortened the climbing time in MPTP-treated mice (Figures 7a and b; *P*<0.05). These results suggest that Nedd4-2 knockdown can ameliorate the movement disorder in PD mice. Furthermore, Nedd4-2 knockdown reversed the reduced number of TH-positive neurons in the SN and TH density in the striatum after MPTP administration (*P*<0.01 and *P*<0.05, respectively; Figures 7c and d). These results indicate that Nedd4-2 knockdown confers neuroprotective effects in PD model.

To demonstrate the role of Nedd4-2 knockdown in the astrocytic activation and microgliosis reported in MPTP model,^43, 44^ we examined the astroglial and microglial expression in SN. Here we found that MPTP induced astrogliosis and microglial activation (Figures 7e and f; *P*<0.01), and Nedd4-2 knockdown attenuated the astrogliosis and activation of microglia in MPTP model (Figures 7e and f; *P*<0.01). Thus we conclude that Nedd4-2 knockdown attenuates astrogliosis and reactive microgliosis in the MPTP model that may be associated with glutamate excitotoxicity.

### Nedd4-2 regulates SGK and PKC signaling in the PD model

To elucidate signaling pathways that may contribute to the neuroprotective effects of Nedd4-2 knockdown in PD model, we examined potential substrates of Nedd4-2 with downstream neuroprotective functions. The E3 family member Nedd4-1 mediates the ubiquitination of *α*-synuclein and exerts neuroprotective effects in PD.^45, 46, 47^ Therefore, we examined whether Nedd4-2 may have a similar effect as Nedd4-1 on *α*-synuclein aggregation in the SN. However, the MPTP-mediated aggregation of *α*-synuclein in the SN was not reduced by Nedd4-2 knockdown (Supplementary Figure 9). These results suggest that *α*-synuclein is not a substrate of Nedd4-2 in PD model.

**Figure 8:**
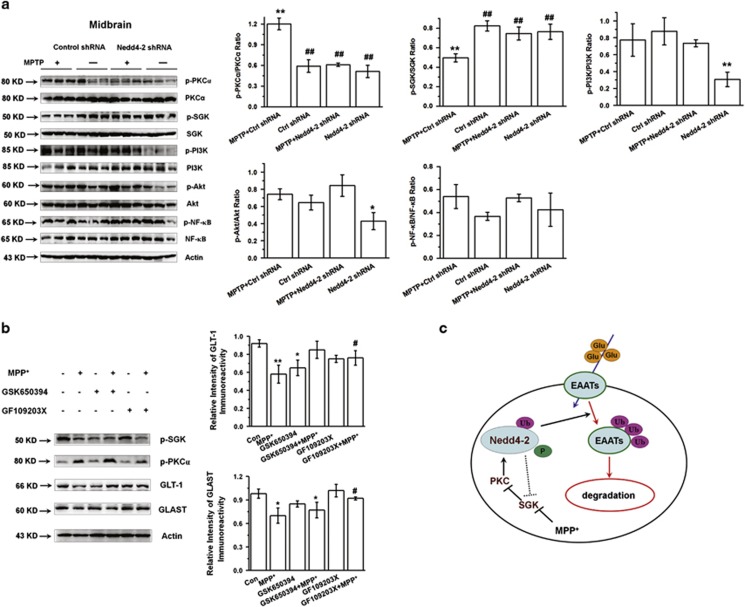
Nedd4-2 regulates the SGK/PKC pathway. (**a**) Western blotting showing that Nedd4-2 knockdown increases p-SGK expression and decreases p-PKC expression in the midbrain of MPTP-treated mice. Results are expressed as the mean±S.E. One-way ANOVA. **P<0.01, *P<0.05, ^##^*P*<0.01, *represents a significant difference between other group and ctrl shRNA group; while ^#^represents a significant difference between the indicated group and MPTP+ctrl shRNA group. *n*=3 per group. (**b**) Western blotting showing that PKC inhibitor increased GLT-1 and GLAST expression in MPP^+^-treated astrocytes. Results are expressed as the mean±S.E. of at least three experiments. One-way ANOVA. **P<0.01, *P<0.05, ^#^*P*<0.05. *Represents a significant difference between other group and control group; while ^#^represents a significant difference between the indicated group and MPP^+^-treated astrocytes for 24 h. (**c**) Model for Nedd4-2 signaling in PD

To explore additional potential downstream signaling pathways, we examined several signal molecules that are known to mediate Nedd4-2 function. Previously, SGK and PKC are thought to regulate Nedd4-2 activity, and PKC can promote the Nedd4-2-mediated ubiquitination of GLT-1.^27, 29^ Furthermore, SGK interacts with Nedd4-2 and abrogates its activity by enhancing the glutamate transporter membrane levels and transport rate.^29^ Consistent with a role for these kinases in mediating Nedd4-2 activity, we demonstrate that MPTP increased the phosphorylation of PKC*α* in the midbrain of mice, but that Nedd4-2 knockdown inhibited the increase (Figure 8a, first two rows). Furthermore, MPTP decreased the phosphorylation of SGK, but Nedd4-2 knockdown inhibited this decrease (Figure 8a, third and fourth row). In the absence of MPTP, Nedd4-2 knockdown also decreased the expression of phosphorylated PI3K and Akt (Figure 8a). Our results show that PKC inhibitor (GF109203X) increased GLT-1 and GLAST expressions in MPP^+^-treated astrocytes (Figure 8b; *P*<0.05), which is consistent with previous studies.^27, 29^ These results suggest that PKC and SGK are regulated downstream of Nedd4-2 in MPTP-treated mice, and that PI3K and Akt are regulated downstream of Nedd4-2 only in the absence of MPTP. Overall, our findings support a model in which Nedd4-2 regulates glutamate transporter function to modulate the effects of PD, and SGK and PKC reciprocally regulate Nedd4-2 activity (Figure 8c).

## Discussion

Glutamate excitotoxicity mediated by dysfunctional glutamate transporters plays an important role in the degeneration of DA neuron. Hence, regulating glutamate transporters is an important issue in the pathogenesis of PD. Herein, we focused on the ubiquitin-proteasome system and the potential role of the E3 ubiquitin ligase Nedd4-2 in PD. Previous studies have demonstrated that the substrates of Nedd4-2 include ENaC, Na_v_s, and voltage-gated potassium channels.^31^ In addition, glutamate transporters have been reported to act as substrates of Nedd4-2.^27, 28, 29, 30^ In addition, PKC regulates the phosphorylation of Nedd4-2 and promotes the endocytosis of GLT-1.^27^ However, before the present study it was not known whether Nedd4-2 regulates the ubiquitination of glutamate transporters and whether Nedd4-2 could comprise a therapeutic target in PD.

Here we report that reducing Nedd4-2 activity is beneficial to PD. We demonstrated that GLT-1 rather than GLAST is ubiquitinated in MPP^+^-treated astrocytes and the interaction between Nedd4-2 and GLAST was not changed after treatment with MPP^+^ or PMA. However, we found that Nedd4-2 interacting with GLAST and Nedd4-2 knockdown increased GLAST expression at total and membrane level in SN *in vivo*, suggesting Nedd4-2 also mediated ubiquitination of GLAST *in vivo*. As Nedd4-2 has also been reported to regulate GLAST,^28^ and moreover, neural activity can regulate GLT-1 and GLAST in astrocyte-neuron coculture system.^48^ We conclude that Nedd4-2 maybe regulated by neural activity and thus the interaction between Nedd4-2 and GLAST shows different pattern in *in vivo* and in only astrocyte-culture system.

Glutamate transporters are shown to contain PKC- or PKA-dependent protein phosphorylation consensus sites. Previously, Casado *et al.* have reported that PMA activate glutamate uptake rate in primary glial cell cultures,^49^ and these authors further provide evidence that PMA-induced activation of glial uptake may be due to a direct phosphorylation of GLT-1 at one of the previously determined PKC phosphorylation sites.^50^ However, PMA treatment for 30 min or much longer promotes the Nedd4-2 mediated-GLT-1 degradation, and this process is PKC-dependent ubiquitination of GLT-1 rather than phosphorylation.^27^ Here in our hand, PMA treatment <30 min increased glial glutamate uptake, while treatment more than 9 h decreased glial glutamate uptake. We speculate that PKC activation in short time promotes GLT-1 via phosphorylation, while in long-time decreases GLT-1 via Nedd4-2-mediated ubiquitination.

Internalized GLT-1 is reported to be colocalized with EEA1, a marker of early endosomes.^33^ It has been suggested that Nedd4-2 binds through its WW domains to the PY motifs of the targeted membrane protein, leading to the membrane protein ubiquitination, endocytosis to endosomes and multivesicular bodies, and degradation in the lysosome.^51^ Besides, Susarla *et al.*^37^ also report that GLT-1 is targeted for lysosomal degradation with prolonged activation of PKC. In this study, we also found that Leupeptin but not MG-132 increased glutamate uptake and GLT-1 expression in MPP^+^-treated astrocytes. Thus we provide evidence that Nedd4-2 may target GLT-1 for ubiquitination, endocytosis to EEA1 and degradation in the lysosome but not proteasome in MPP^+^-treated astrocytes.

We also demonstrate that GLT-1 expression and glutamate uptake in the synaptosomes are decreased at 5+3d of MPTP administration. However, in the animal experiments, homeostatic range of the organism can indeed influence the glutamate uptake in the synaptosome. Actually, the onset of motor symptoms in PD animal models has been shown to be closely tied with the increase of glutamate levels within the basal ganglia,^52, 53^ and we also found that behavioral disorder and decreased TH expression are consistent with decreased glutamate uptake in the synaptosome of PD model (Figures 4o and r; Supplementary Figure 3). The variation in glutamate uptake is also proved by reduced glutamate transporters protein expression in PD model (Figures 3c and f). Moreover, the decreased glutamate uptake in the synaptosome at 5+1d of MPTP administration also proves that such level of variation is not a fictional effect (Figures 4n and q).

In this study, we for the first time show that Nedd4-2 knockdown ameliorates the movement disorder in PD model and increases the percentage of TH-positive neurons in the SN and TH density in striatum. These results suggest that Nedd4-2 knockdown is neuroprotective in PD mice. Because glutamate excitotoxicity can induce DA neuron death in PD models,^52, 54^ and we demonstrated that Nedd4-2 knockdown increases TH expression, we conclude that this neuroprotection is explained by the regulation of glutamate transporters. Previous studies indicate that MPTP lesion can induce remarkable astrocytic activation and robust microgliosis via glutamate excitotoxicity in SNpc.^43, 44^ For the first time, we report that Nedd4-2 knockdown attenuates astrogliosis and reactive microgliosis in MPTP model. We also showed that Nedd4-2 knockdown decreases phosphorylated PKC*α* expression and increases phosphorylated SGK expression in the midbrain. In addition, PKC has been shown to promote Nedd4-2-mediated ubiquitination of GLT-1.^27, 29^ Here we also indicate that PKC suppression increase GLT-1 and GLAST expression. Therefore, these findings suggest that Nedd4-2 knockdown alters the activity of SGK and PKC, possibly in a feedback loop, which could impact its function (Figure 8c).

Taken together, we report that Nedd4-2 mediates the ubiquitination of glutamate transporters in *in vitro* and *in vivo* of PD models. Nedd4-2 knockdown improves the motor deficits and TH expression in PD mice via increasing glutamate transporters, and for the first time, we report that Nedd4-2 knockdown attenuates astrogliosis and reactive microgliosis in MPTP model. Thus Nedd4-2 should be considered as a potential therapeutic target for the treatment of PD.

## Materials and Methods

### Reagents

l-[^3^H]-Glutamic acid was purchased from PerkinElmer (Boston, MA, USA). MPP^+^ and MPTP were purchased from Sigma-Aldrich (St. Louis, MO, USA). Dulbecco's modified Eagle medium/F12 (DMEM/F12) and fetal calf serum were purchased from Hyclone (Logan, UT, USA). Anti-GLT-1 monoclonal antibody and anti-TH antibody were purchased from Millipore (Bedford, MA, USA). Antibodies against Nedd4-2, phospho-Nedd4-2-Ser342 and GFAP were purchased from Cell Signaling Technology (Danvers, MA, USA). Anti-ubiquitin antibody, EEA1 antibody, Iba-1 antibody and protein A/G agarose were purchased from Santa Cruz Biotechnology (Santa Cruz, CA, USA). Anti-phospho- PKC*α* (Thr638) antibody, anti-PKC*α* antibody, anti-phospho-SGK (Ser422) antibody, anti-SGK antibody, anti-phospho-PI3 kinase p85-*α* (Tyr607) antibody, anti-PI3 kinase p85-*α* antibody, anti-phospho-Akt (Ser473) antibody, anti-Akt antibody, anti-phospho-NF-*κ*B p65 (Ser276) antibody, anti-NF-*κ*B p65 antibody and integrin antiobody were purchased from EnoGene (Nanjing, China). Anti-actin antibody and normal mouse and rabbit IgG were purchased from Beyotime (Shanghai, China). FITC-conjugated goat anti-mouse antibody, TRITC-conjugated goat anti-rabbit antibody, and HRP-conjugated goat anti-mouse and rabbit antibody were purchased from Boster (Wuhan, China). EZ-Link Sulfo-NHS-SS-Biotin was purchased from Thermo Scientific (#21331, Waltham, MA, USA). Trizol was purchased from Invitrogen (Carlsbad, CA, USA). PrimeScript RT reagent kits and SYBR Premix Ex Taq kits were purchased from Takara (Otsu, Japan). SGK inhibitor (GSK650394), PKC inhibitor (GF109203X), proteasome inhibitor (MG-132) and lysosome inhibitor (Leupeptin) were purchased from Selleck (Houston, TX, USA).

### Cell culture

Primary cortical astrocytes were prepared as described previously.^22^ Primary astrocytes were cultured in DMEM/F12 supplemented with 10% fetal calf serum and equilibrated in humidified air containing 5% CO_2_ at 37 °C.

### Animals

Adult (10-week-old, male) C57BL/6 mice were obtained from the Guangdong Medical Animal Laboratory (Foshan, China). Three mice per cage were housed with a 12:12- h light/dark cycle and had free access to food and water. They were allowed to adapt to the environment for at least 3 days before experiments.

### Nedd4-2 siRNA transfection in MPP^+^-treated astrocytes

Three target Nedd4-2 mRNAs were designed and synthesized by RioBio (Guangzhou, China). The three target sequences were as follow: siRNA-1, 5′-GCCAUCAGUGGCCUAUGUA-3′ siRNA-2, 5′-GCAGAAAUACGACUACUUU-3′ and siRNA-3, 5′-GGUCCUCAGCUGUUUACAA-3′. The negative control siRNA (scrambled siRNA) was also provided by RioBio. Transfection was performed according to the manufacturer's instructions. In brief, primary astrocytes were seeded into the 24-well plates or 6-cm dishes. SiRNA stock solution was diluted to the working solution (100 nM) using riboFECT CP Buffer (RioBio, Guangzhou, China). The cells were incubated with riboFECT CP Reagent (RioBio) for 15 min, and then the working solution was added into the culture medium and the cells were incubated for 72 h. Nedd4-2 protein expression was detected by western blotting after transfection for 72 h.

In subsequent experiments, cells were randomly divided into four groups: (1) the control group: cells were pretreated with phosphate-buffered saline (PBS) for 72 h and then incubated with normal culture medium for 24 or 48 h; (2) the Nedd4-2 siRNA group: cells were pretreated with Nedd4-2 siRNA for 72 h and then incubated with normal culture medium for 24 or 48 h; (3) the MPP^+^ group: cells were pretreated with PBS for 72 h and then incubated with MPP^+^ for 24 or 48 h; (4) the MPP^+^+Nedd4-2 siRNA group: cells were pretreated with Nedd4-2 siRNA for 72 h and then incubated with MPP^+^ for 24 or 48 h. The addition of MPP^+^ to astroglial cultures was performed at 1 mM as stated by our work and others,^22, 55^ and the culture meduim containing half-serum was used to dilute MPP^+^.

### Inhibitors treatment

To determine whether reduction in GLT-1 expression due to ubiquitination can be reversed by proteasome or lysosome inhibition, cells were divided into control and MPP^+^ group, and in the meanwhile, these two groups were incubated with proteasome inhibitor (1 *μ*M MG-132) or lysosome inhibitor (5 *μ*M Leupeptin) for 24 h. To determine whether SGK and PKC participate in Nedd4-2 regulating glutamate transporters, cells were divided into control and MPP^+^ group, and in the meanwhile, these two groups were incubated with SGK inhibitor (10 *μ*M GSK650394) or PKC inhibitor (1 *μ*M GF109203X) for 24 h. Then the glutamate uptake and glutamate transporters expression were examined.

### MPTP treatment of mice

For generating the PD animal model, C57BL/6 mice received one intraperitoneal injection of MPTP-HCl daily (30 mg/kg free base in saline) for 5 days and were killed at the indicated time points.

### Stereotaxic injection of Nedd4-2 shRNA in SN

Lentivirus vector (LV)-sh[NEDD4-2] and LV-sh[control] were generated by ligating annealed oligonucleotides encoding shNedd4-2 (see the Supplementary Figure 8a) or a control sequence into the *Bam*H I/*Eco*R I sites of pHBLV-U6-ZsGreen-Puro vector. LV-sh[NEDD4-2] was constructed to express shRNA targeting *Nedd4-2* from the U6 (RNA polymerase III) promoter with PGK-driven puromycin resistance and CMV IE-driven ZsGreen expression. LV-shNedd4-2 was constructed and the virus was packaged by HanBio (Shanghai, China). Negative control shRNA (scrambled shRNA) was kindly gifted by HanBio (Shanghai, China). C57BL/6 mice were maintained in a temperature/humidity controlled environment with free access to food and water.

For *in vivo* viral injections, mice were anesthetized and placed in a stereotaxic frame. LV-Nedd4-2 shRNA or control shRNA viral vectors were stereotaxically injected into the right side of the pars compacta of substantia nigra at the target site (Bregma AP, −3.0 mm, ML, +1.3 mm, DV, −4.7 mm). In brief, a Hamilton syringe was filled with LV-Nedd4-2 shRNA or control shRNA virus and the needle was lowered into the tissue at a rate of 0.5 *μ*l/min. The syringe was left in place for 5 min before being slowly withdrawn from the brain. To obtain the optimal inhibitory efficiency, we injected 2.0, 3.0, or 4.0 *μ*l of virus into the SN, and at 3, 5, or 7 days after injection, mice were sacrificed for dissection of the right side of the midbrain for Nedd4-2 quantification by western blotting. In subsequent experiments, we injected 3.0 *μ*l of virus into the SN, and 5 days after injection we intraperitoneally injected MPTP-HCl (30 mg/kg free base in saline) or saline for another 5 days. Three days after the last MPTP injection, behavioral tests were performed and then the mice in each group were killed for the indicated experiments.

### Behavioral tests

The behavioral tests were performed as stated previously.^56, 57^ (1) Grasping test. Mice were assessed using the grasping experiment 3 days after the last MPTP injection. In brief, mice were suspended by their forelimbs on a metal rod (diameter: 1.5 mm) and positioned ~30 cm above the box. The holding time on the metal rod was recorded; (2) Pole-climbing test. Mice were placed on the peak of a foam ball (diameter: 2.0 cm) fixed on the stick (diameter: 1.0 cm, length: 50 cm). The climb time from the peak to the bottom of the stick was recorded.

### Tissue preparation

After behavioral tests, the mice in each group were perfused to obtain brain sections. The mice were anesthetized with intraperitoneal injection of chloral hydrate and then perfused transaortally with 60 ml of 0.9% saline to remove any blood followed by 60 ml of 4% paraformaldehyde. Subsequently, the fixed brains were immediately removed from the skulls, stored overnight at 4 °C in 4% paraformaldehyde, and dehydrated in a gradient of 20–30% sucrose. The brain samples were embedded in optimum cutting temperature compound (Sakura Finetek, Torrance, CA, USA) and then cut into 10 *μ*m sections with a freezing microtome (Lecia, Germany). All sections were stored at −80 °C for immunohistochemistry and immunofluorescence staining. For other experiments, at the 3rd day of MPTP injection (3d), the 1st day after the last MPTP injection (5+1), or the 3rd day after the last MPTP injection (5+3), mice were anesthetized with intraperitoneal injection of chloral hydrate and then perfused transcardially with 60 ml of 0.9% saline to remove traces of blood. The midbrain and striatum tissues were removed and stored at −80 °C until further assessment.

### Immunohistochemistry assay

Sample sections were cut into 10 *μ*m slices and antigen retrieval was performed using citrate buffer. The slices were permeabilized with 0.3% Triton X-100 and blocked with bovine serum albumin (BSA) for 10 min. Then the slices were incubated with primary antibodies overnight at 4 °C. After washing with PBS, the slices were incubated with secondary antibodies for 2 h at 37 °C. Antibody-peroxidase complexes were revealed by incubating the slices with 3,3-diaminobenzidine peroxidase substrate (Boster, Wuhan, China). Total numbers of TH-positive neurons in SNpc in Figure 7c and Supplementary Fig. 3c were counted stereologically using the optical fractionator method.^58^ Striatal OD of TH immunostaining in Figure 7d and Supplementary Figure 3d determined by the Image-Pro Plus 6.0 photogram analysis system (IPP 6.0, Media Cybernetics, Bethesda, MD, USA), was used as an index of striatal density of TH innervation.^59^ TH-positive neurons number and integrated OD was assessed at 400 × magnification in at least 10 high-power fields from brain sections of representative mice in each group.

### L-[^3^H]-Glutamic acid uptake assay

Astrocytes were seeded in 24-well plates. The L-[^3^H]-Glutamic acid uptake assay was performed as described previously.^22^ After washing once with choline solution (150 mM choline chloride, 5 mM KP_i_, pH 7.4, 0.5 mM MgSO_4_ and 0.3 mM CaCl_2_), 0.4 *μ*Ci/well L-[^3^H]-Glutamic acid (specific activity 12.9 Ci/mmol) was added to the wells. The cells were incubated for 10 min at room temperature and the reaction was stopped by the two applications of ice-cold NaCl solution (150 mM NaCl, 5 mM KP_i_, pH 7.4, 0.5 mM MgSO_4_ and 0.3 mM CaCl_2_). 1% SDS was added to lyse the cells, and the radioactivity was measured by liquid scintillation counting. Data are derived from three separate experiments performed in triplicate.

### Determination of glutamate uptake in the synaptosomes

Midbrain and striatum tissues were homogenized in 0.32 M sucrose solution (0.32 M sucrose, 5 mM Hepes, pH 7.4) and centrifuged at 1,000 × *g* for 15 min. The resulting supernatant was centrifuged further at 15 000 × *g* for 30 min, and then the precipitate was resuspended in 0.32 M sucrose solution to obtain the crude synaptosomes. The protein concentrations were determined using a BCA colormetric assay, and crude synaptosomes were resuspended at a final concentration of 0.5 mg/ml in Kreb's buffer (127 mM NaCl, 3.73 mM KCl, 1.8 mM CaCl_2_, 1.18 mM KH_2_PO_4_, 20 mM NaHCO_3_, 2 mM ATP, 2 g/l d-glucose, pH7.4) in a water bath at 25 °C for 10 min. For the reuptake assay, 1 *μ*Ci L-[^3^H]-glutamic acid was added to the synaptosome preparations in a water bath at 25 °C for another 10 min. The reactions were terminated with 5 ml of ice-cold Kreb's buffer. Synaptosomes were washed three times with PBS to remove excess labeled glutamate and then were filtered with 0.22 *μ*m filter paper. The filter papers containing the rinsed synaptosomes were then placed in scintillation vials containing 3 ml of scintillation cocktail in the dark overnight. The radioactivity was measured on the next day by liquid scintillation counting. The glutamate uptake in the synaptosomes was measured as cpm/mg protein/min. Data are from three separate experiments, and each experiment was performed in triplicate.

### Real-time qPCR

Extraction of total tissue or cellular RNA was performed according to the manufacturer's instructions. Then the RNA was reverse-transcribed to synthesize first-strand cDNA, and the cDNA was quantified using the SYBR Premix Ex Taq kit. For each transcript, a mixture of the following reaction components was prepared to the indicated end-concentration: forward primer (10 *μ*M), reverse primer (10 *μ*M), and SYBR Green Premix Ex Taq. The fluorescence was detected on a Corbett research RG-6000 real-time PCR machine (Corbett Life Science, Sydney, Australia). The primer sequences were as follows: GLT-1 (forward) 5′-CGATGAGCCAAAGCACCGAA-3′, (reverse) 5′-CTGGAGATGATAAGAGGG AGGATG-3′; GLAST (forward) 5′-TCAAGTTCTGCCACCCTACC-3′, (reverse) 5′-TCTGTCCAAA GTTCAGGTCAA-3′; *β*-actin (forward) 5′-CTACAATGAGCTGCGTGTGGC-3′, (reverse) CAG 5′-GTCCAGACGCAGGATGGC-3′. Each sample was compared with Actin as the internal control. Data are from three separate experiments, and each performed in triplicate.

### Colocalized immunofluorescence assay

Brain tissue slices from each group were fixed in 4% paraformaldehyde and then rinsed with PBS. Afterwards, samples were permeabilized with 0.1% Triton X-100 and blocked with 5% BSA. For the colocalization study, the slices were incubated with primary antibodies (GLT-1 or GALST and Nedd4-2) overnight at 4 °C, rinsed with PBS and incubated with FITC-conjugated goat anti-mouse IgG and TRITC-conjugated goat anti-rabbit IgG for 2 h at 37 °C. DAPI was used to stain cell nuclei. Immunostaining was then examined using an Olympus 1 × 81 FV1000 Laser Scanning Confocal Microscope (Shinjuku, Tokyo, Japan). Quantification of double-labeled immunecytological antigens that were colocalized was performed by imaging and analyzing cells using IPP 6.0 (media Cybemetics, Bethesda, MD, USA). Generally, GLT-1 or GLAST clusters were selected automatically in the pseudo-colored ‘red' channel as discrete puncta of intensity >1.5-fold brighter than the background fluorescence. Selected clusters were transferred to the green channel to measure the Nedd4-2 fluorescence. Colocalization of Nedd4-2 over GLT-1 or GLAST was measured as the percentage of integrated Nedd4-2 pixel intensities that overlapped with the GLT-1 or GLAST fluorescence in individual clusters. As a negative control, the primary antibody was replaced with 5% BSA.

### Co-IP assays

Brain tissues or primary astrocytes were lysed in ice-cold RIPA buffer. Antibodies were incubated with the lysates overnight at 4 °C and then with protein A/G agarose beads for 4 h at 4 °C. The beads containing antigen-antibody complexes were washed with RIPA buffer four times. The proteins on the beads were extracted with 60 *μ*l SDS sample buffer. Samples were immediately boiled at 95 °C for 5 min before Western blotting assay. The normal mouse or rabbit IgG was set as the negative control.

### Total protein extraction

For the total membrane protein extraction, cells or brain tissues were lysed with radio-immunoprecipitation assay (RIPA) buffer (Beyotime, Shanghai, China) containing 1 mM PMSF (Beyotime, Shanghai, China). Protein concentrations were measured by BCA assay. Samples were diluted with protein loading buffer and heated to 95 °C for 5 min prior to western blotting.

### Cell surface biotinylation

Cell surface expressions of glutamate transporters were examined using the membrane-impermeable biotinylation reagent EZ-Link Sulfo-NHS-SS-Biotin. Cells or tissues were washed twice with ice-cold PBS (pH 8.0) and then incubated with 2.5 ml of EZ-Link Sulfo-NHS-SS-Biotin (0.5 mg/ml in PBS) in two successive 20 min incubations on ice with gentle shaking. Then cells or tissues were washed twice with 100 mM glycine to remove non-reacted biotinylation reagent. Cells or homogenized tissues were lysed on ice for 20 min in 750 *μ*l of cell lysis buffer containing the protease inhibitor mixture and 1 mM PMSF. After centrifugation at 12 000 × *g* for 20 min, the debris was removed. Supernatants were transferred to new tubes and 200 *μ*l of streptavidin-agarose beads were added to bind the biotin-labeled cell membrane proteins. Centrifugation for 1 min at 4 °C and discard supernatant, then washing three times with ice-cold lysis buffer. At last washing once with ice-cold PBS and centrifugation for 1 min at 4 °C. The lower are the member protein sample and are used for western blotting.

### Western blotting assay

Protein samples were resolved via 12% SDS-PAGE and then were transferred to polyvinylidene difluoride membranes. The membranes were blocked with 5% BSA for 2 h at room temperature and were incubated with primary antibodies overnight at 4 °C. Then the membranes were washed three times with TBS-T (Tris-buffered saline containing 0.1% Tween 20) and incubated with HRP-conjugated secondary antibodies. Enhanced chemiluminescence (Millipore, MA, USA) was used to visualize the proteins in the membrane and the chemiluminescent immunoreactive complexes were visualized using a Tanon imager system (Shanghai, China). Protein levels were quantified using Image J software (NIH Image, Bethesda, MD). Actin or integrin immunoreactivity was set as the internal control.

### Statistical analysis

Data are presented as the means±S.E.M. of three independent experiments. Statistical analysis of the data were performed with the Student's *t*-test for two-way comparison or the one-way analysis of variance (ANOVA) test for multiple comparisons using SPSS 16.0 (SPSS Inc, Chicago, IL, USA). Significance was considered as *P*<0.05.

### Study approval

All animal protocols in this study were approved by the Institutional Animal Care and Use Committee of the Southern Medical University.
