# Supplementary material for: Regulation of glutamate transporter trafficking by Nedd4-2 in a Parkinson's disease model
Source: Cell Death Dis. 2017 Feb 2;8(2):e2574–. doi: 10.1038/cddis.2016.454 (PMC5386455; doi:10.1038/cddis.2016.454)
Supplement: Supplementary Figures [file cddis2016454x1.pdf]

Supplemental Material

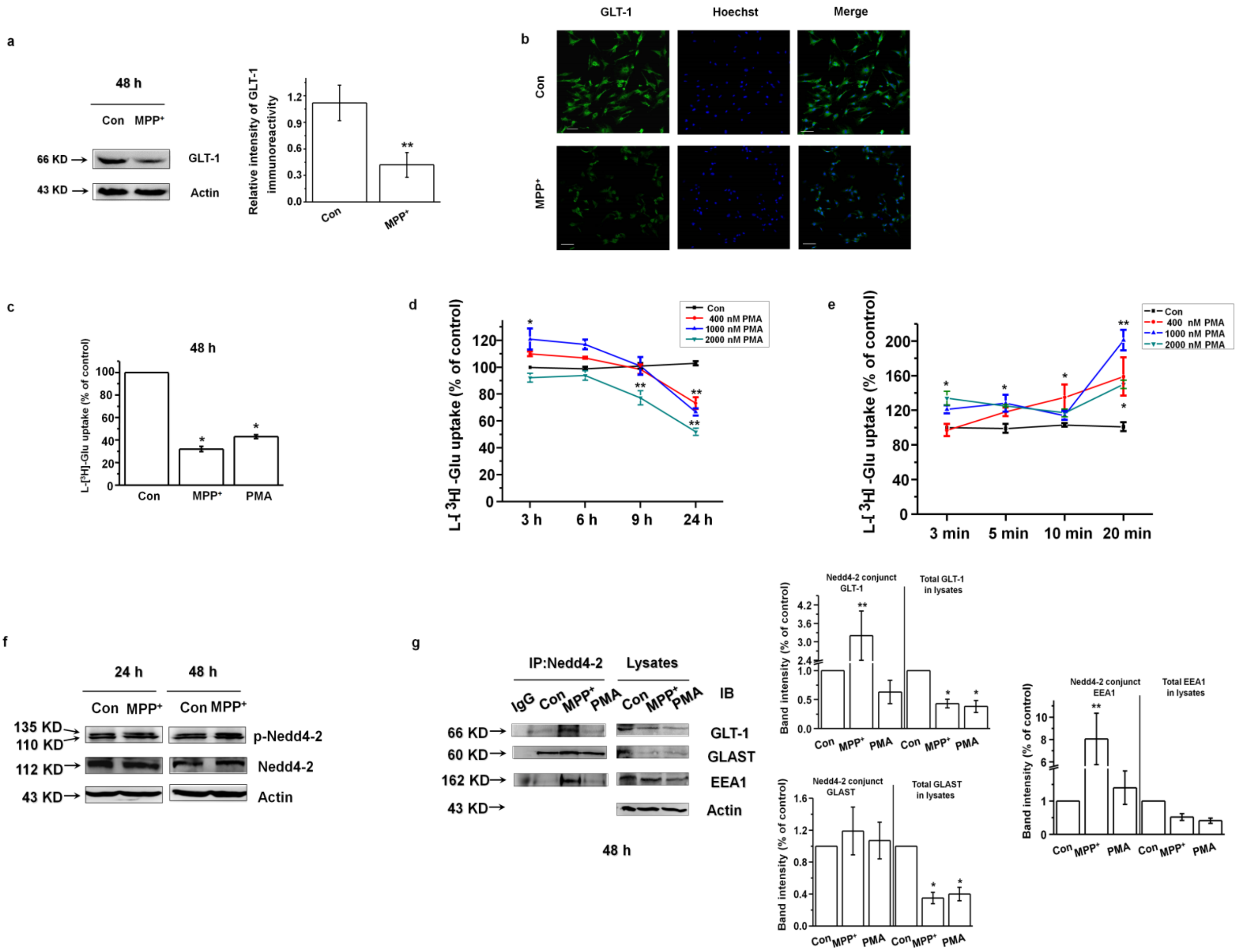

Supplementary Figure 1 (**a**) Western blotting analysis showing that GLT-1 expression is decreased upon MPP$^+$ treatment of astrocytes for 48 h. (**b**) Immunofluorescent staining showing that GLT-1 expression is obviously decreased after 48 h MPP$^+$ treatment in astrocytes. Scale bar: 40 μm. (**c**) L-[^{3}H]-Glutamic acid uptake assay showing that 1 mM MPP$^+$ and 2 μM PMA treatment for 48 h decreases the glutamate uptake in astrocytes. (**d** and **e**) Effects of different concentrations of PMA (500, 1000, 2000 nM) on astrocytes at different time points. Glutamate uptake was decreased after 6 or more hours of PMA treatment (**panel d**, but was increased at shorter time points (3-20 min; **panel e**). (**f**) Western blotting analysis showing that Nedd4-2 expression and phosphorylation does not change significantly after 48 h MPP$^+$ treatment of astrocytes. (**g**) Co-IP assay showing the interaction between Nedd4-2 and GLT-1 in MPP$^+$-treated astrocytes. The capture antibody in **panel g** was anti-Nedd4-2 and MPP$^+$ treatment time was 48 h. Results are expressed as the mean ± SE of at least 3 independent experiments. One-way ANOVA. $^{**}p < 0.01$, $^{*}p < 0.05$ versus control.

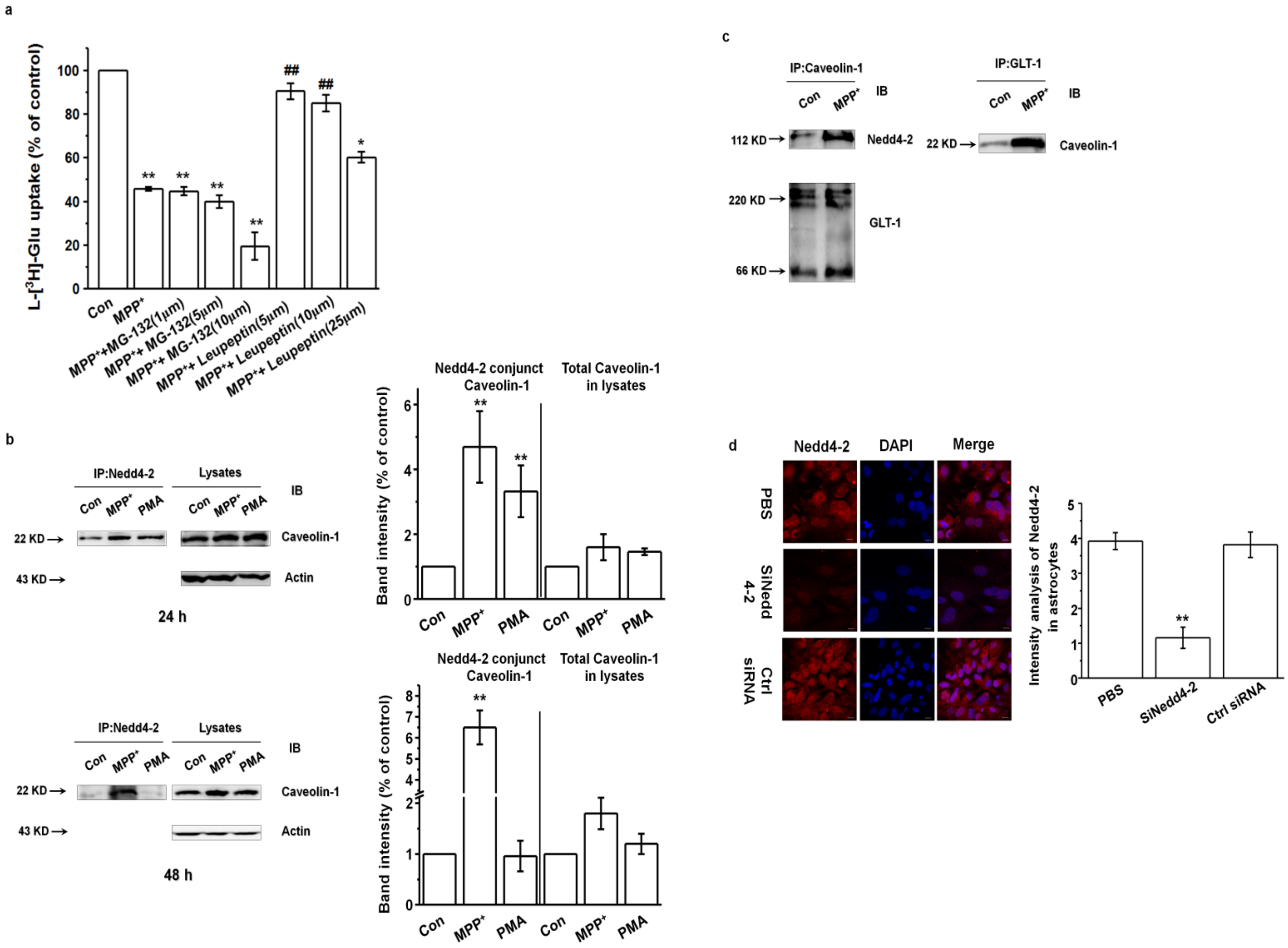

Supplementary Figure 2 (**a**) Glutamate uptake assay showing that different concentration of Leupeptin increased glutamate uptake in MPP^+-treated astrocytes, while MG-132 showed no obvious effects. (**b** and **c**) Co-IP assay showing that Nedd4-2 interacts with caveolin-1 and GLT-1 in MPP^+-treated astrocytes. In **panel b**, the MPP^+ treatment was 24 h and 48 h and the capture antibody was Nedd4-2; in **panel c**, the MPP^+ treatment was 24 h and the capture antibody was caveolin-1 or GLT-1. (**d**) Immunofluorescent staining showing that Nedd4-2 expression was decreased in astrocytes at 72 h after transfection with Nedd4-2 siRNA compared with transfection with PBS or negative control siRNA. Scale bar: 30 μm. Results are expressed as the mean ± SE of at least 3 independent experiments. One-way ANOVA. $^{**}p < 0.01$, $^{*}p < 0.05$ versus control. $^{\#\#}p < 0.01$ versus MPP^+ group.

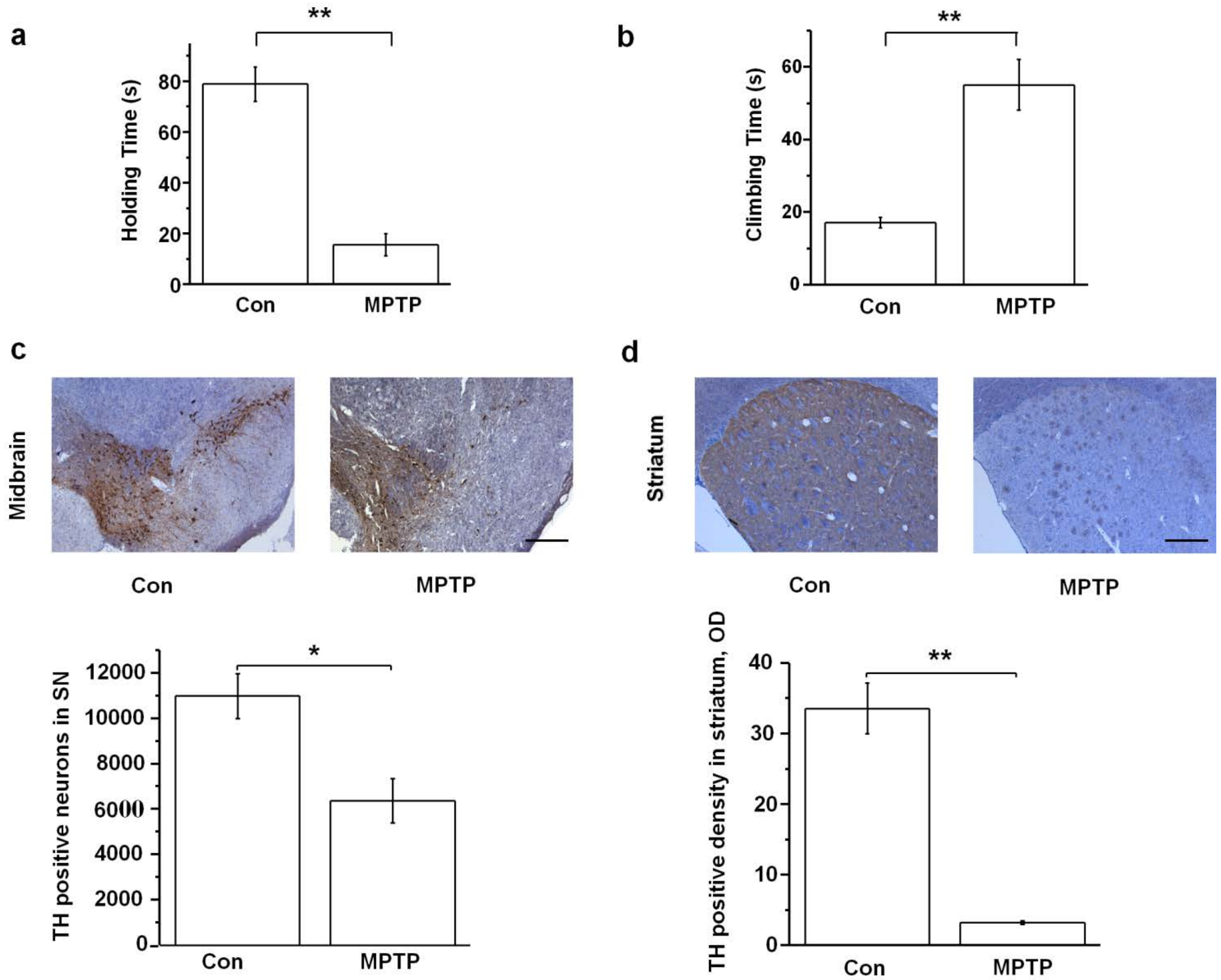

Supplementary Figure 3 Development of a PD mouse model by intraperitoneal injection of MPTP. (**a** and **b**) Behavior tests. The holding time was shortened (**panel a**) and the climbing time was prolonged (**panel b**) in MPTP-treated mice. The results are expressed as the mean ± SE. Student's *t*-test. n = 12 in each group. $^{**}p < 0.01$. (**c** and **d**) IHC staining showed that TH expression is decreased in the SN (**panel c**) and striatum (**panel d**) of MPTP-treated mice. Results are expressed as the mean ± SE. Student's *t*-test. n = 6 per group. $^{*}p < 0.05$, $^{**}p < 0.01$ versus control. Scale bar: 100 μm.

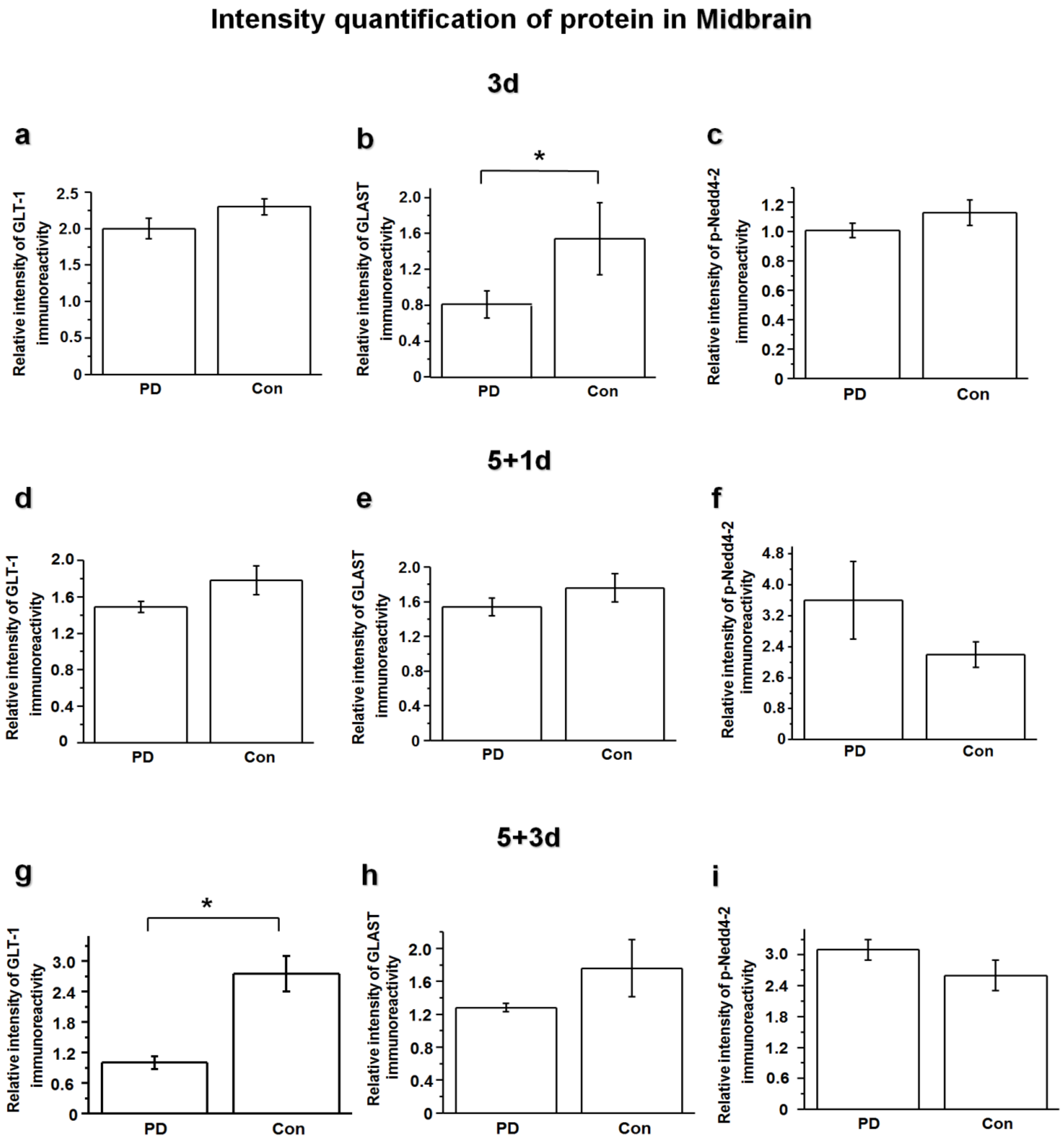

Supplementary Figure 4 The protein expression of glutamate transporters is decreased in the midbrain in MPTP-treated mice. Protein levels in midbrain were quantified using Image J software and actin immunoreactivity was set as the loading control. GLAST expression was decreased in the 3d group (**panel b**), and GLT-1 expression was decreased in the 5+3d group (**panel g**) in the midbrain of MPTP-treated mice. Results are expressed as the mean ± SE. Student's *t*-test. n = 3 per group. $^{*}p < 0.05$ versus control.

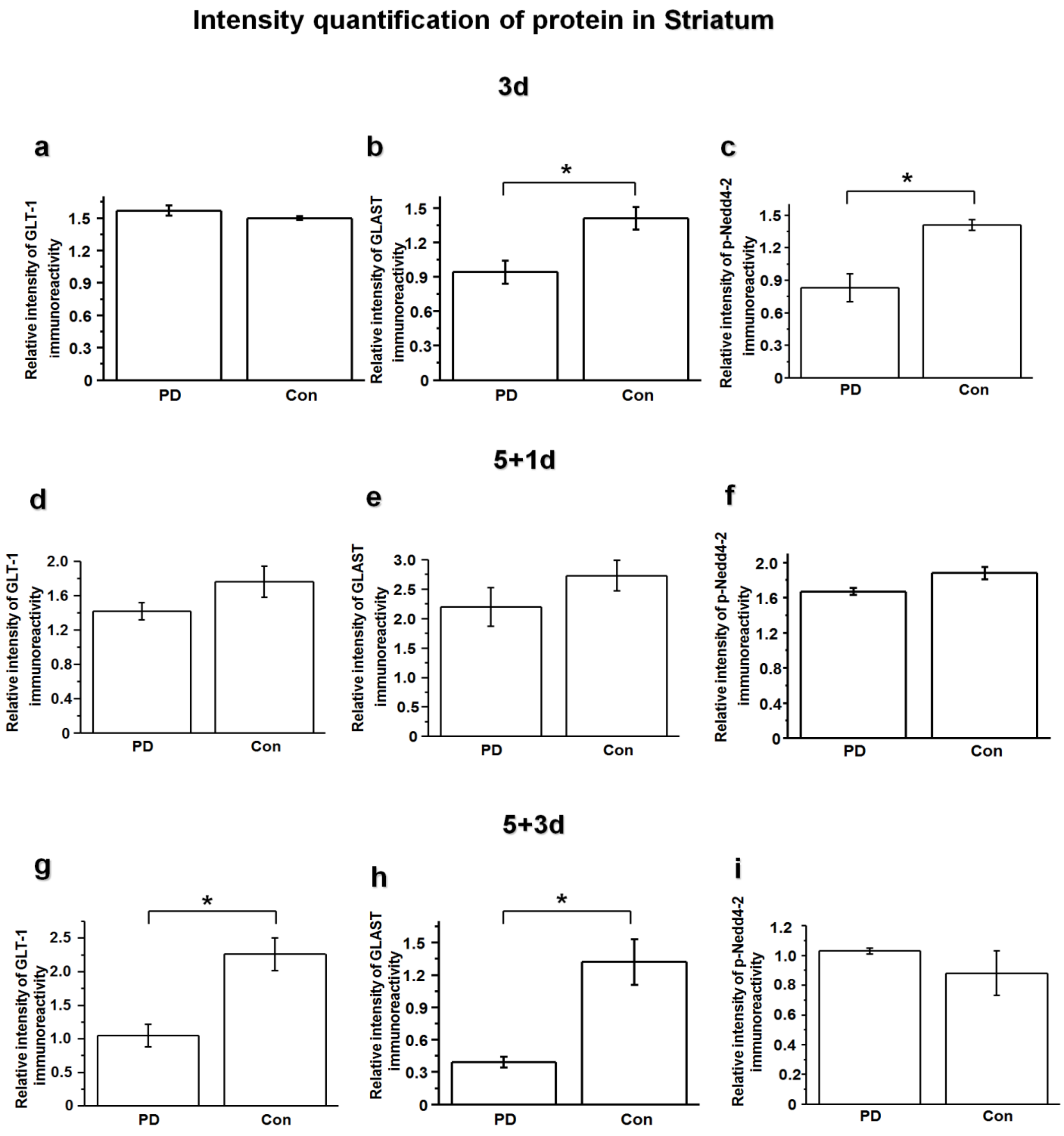

Supplementary Figure 5 The protein expression of glutamate transporters is decreased in the striatum in MPTP-treated mice. Protein levels in striatum were quantified using Image J software and actin immunoreactivity was set as the loading control. GLT-1 expression was decreased in the 5+3d group (**panel g**), and GLAST expression was decreased in the 3d and 5+3d groups (**panel b** and **h**) in the striatum of MPTP-treated mice. p-Nedd4-2/Nedd4-2 expression was decreased in the 3d group (**panel c**) in the striatum of MPTP-treated mice. Results are expressed as the mean ± SE. Student's *t*-test. n = 3 per group. $^{*}p < 0.05$ versus control.

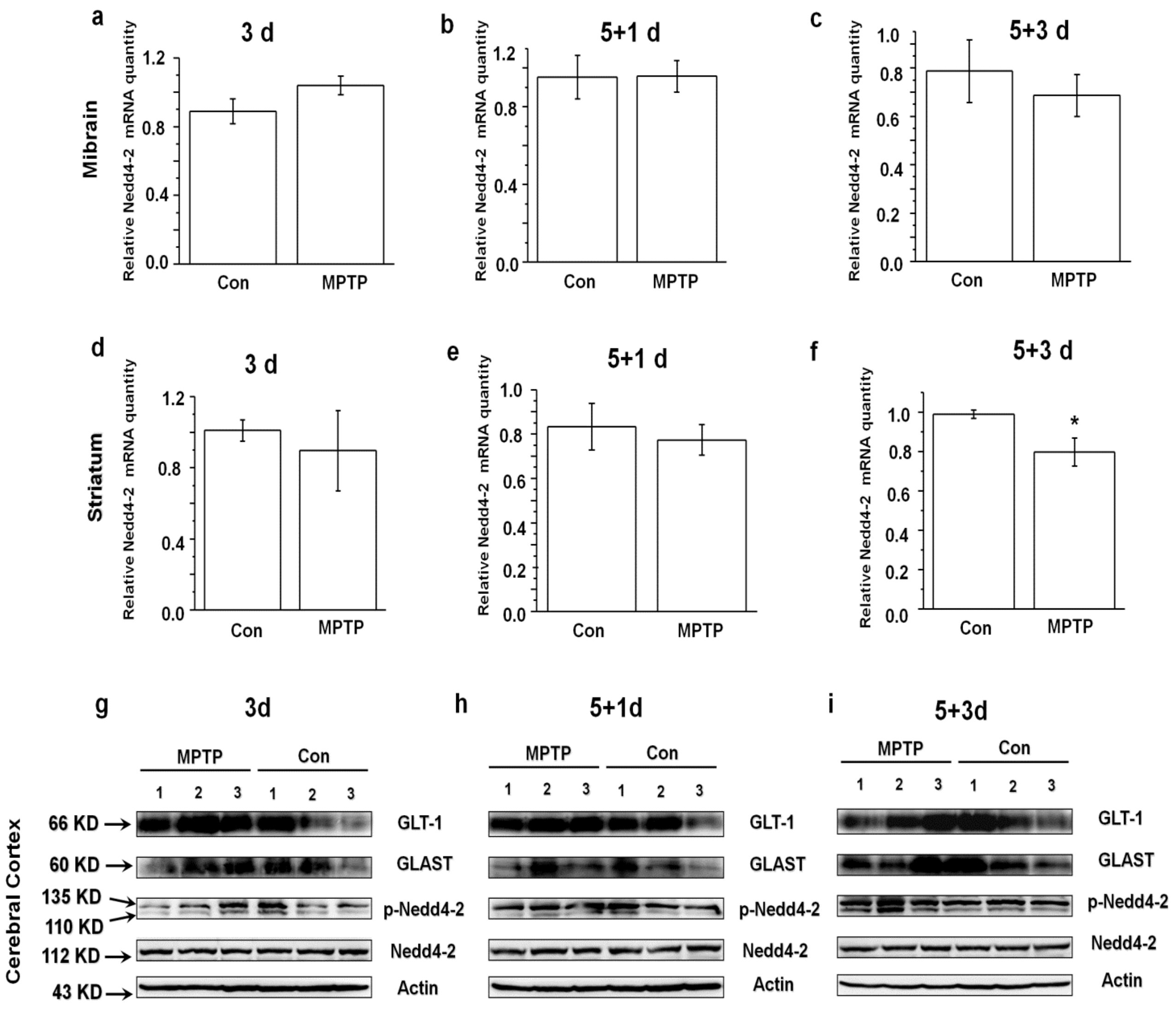

Supplementary Figure 6 Nedd4-2 mRNA expression in the midbrain and striatum of MPTP-treated mice. (**a**–**f**) qPCR assay showing Nedd4-2 mRNA expression in the midbrain and striatum. Nedd4-2 mRNA expression was decreased in the 5+3d group in the striatum (**panel f**). (**g-i**) The protein expressions of glutamate transporters, p-Nedd4-2 and Nedd4-2 in the cerebral cortex were unchanged in MPTP-treated mice. Results are expressed as the mean ± SE. Student's *t*-test. n = 3 per group. $^{*}p < 0.05$ versus control.

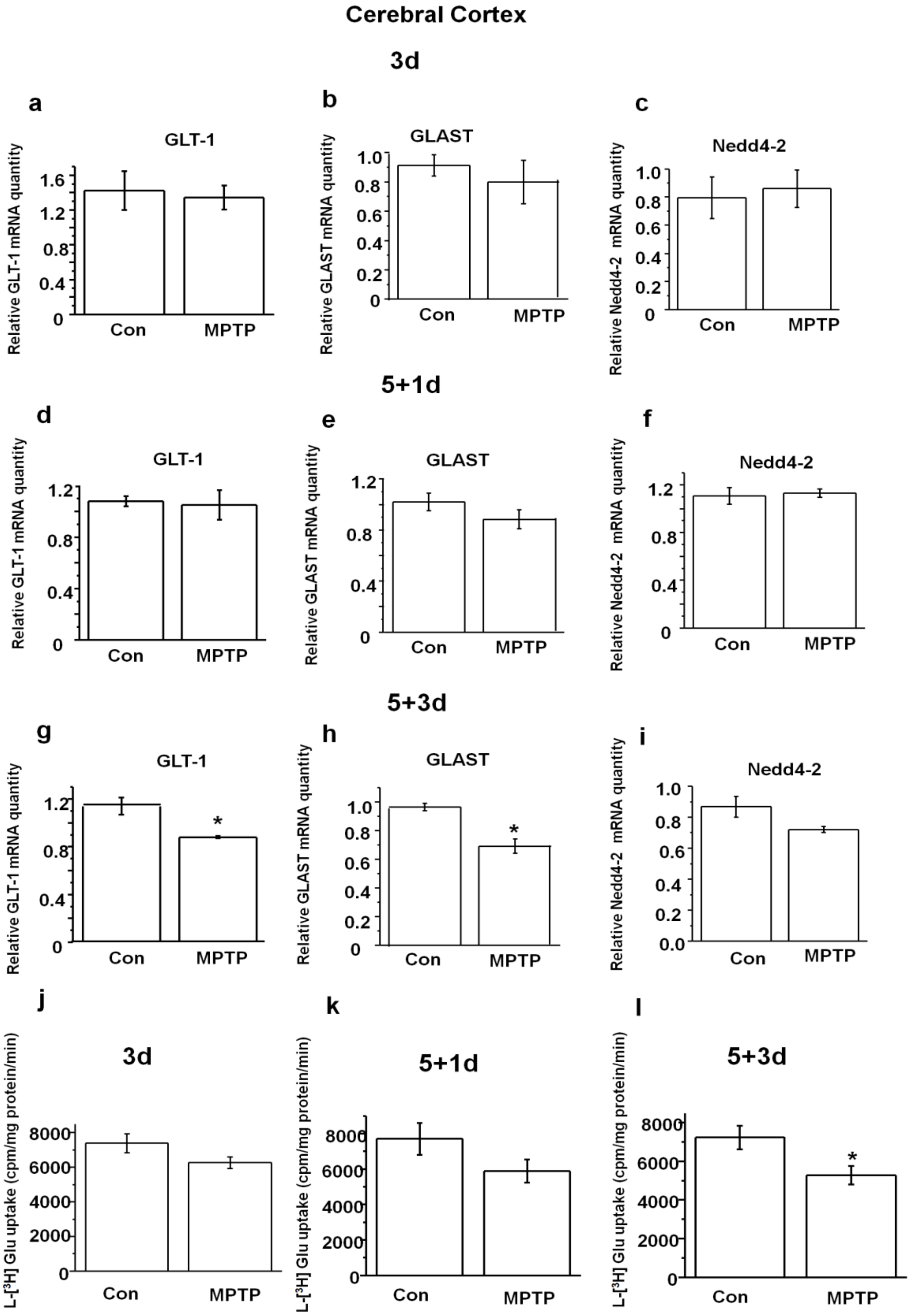

Supplementary Figure 7 Glutamate transporters and Nedd4-2 mRNA expression in cerebral cortex of MPTP-treated mice. (**a–i**) qPCR analysis showing glutamate transporters and Nedd4-2 mRNA expression in cerebral cortex from MPTP-treated mice at different time points. GLT-1 and GLAST mRNA expression was decreased in the 5+3d group in the cerebral cortex (**panel g** and **h**). (**j–l**) The glutamate uptake in the synaptosomes was decreased in 5+3d group in the cerebral cortex in MPTP-treated mice. Student's *t*-test. n = 6 per group. $^{*}p < 0.05$ versus control.

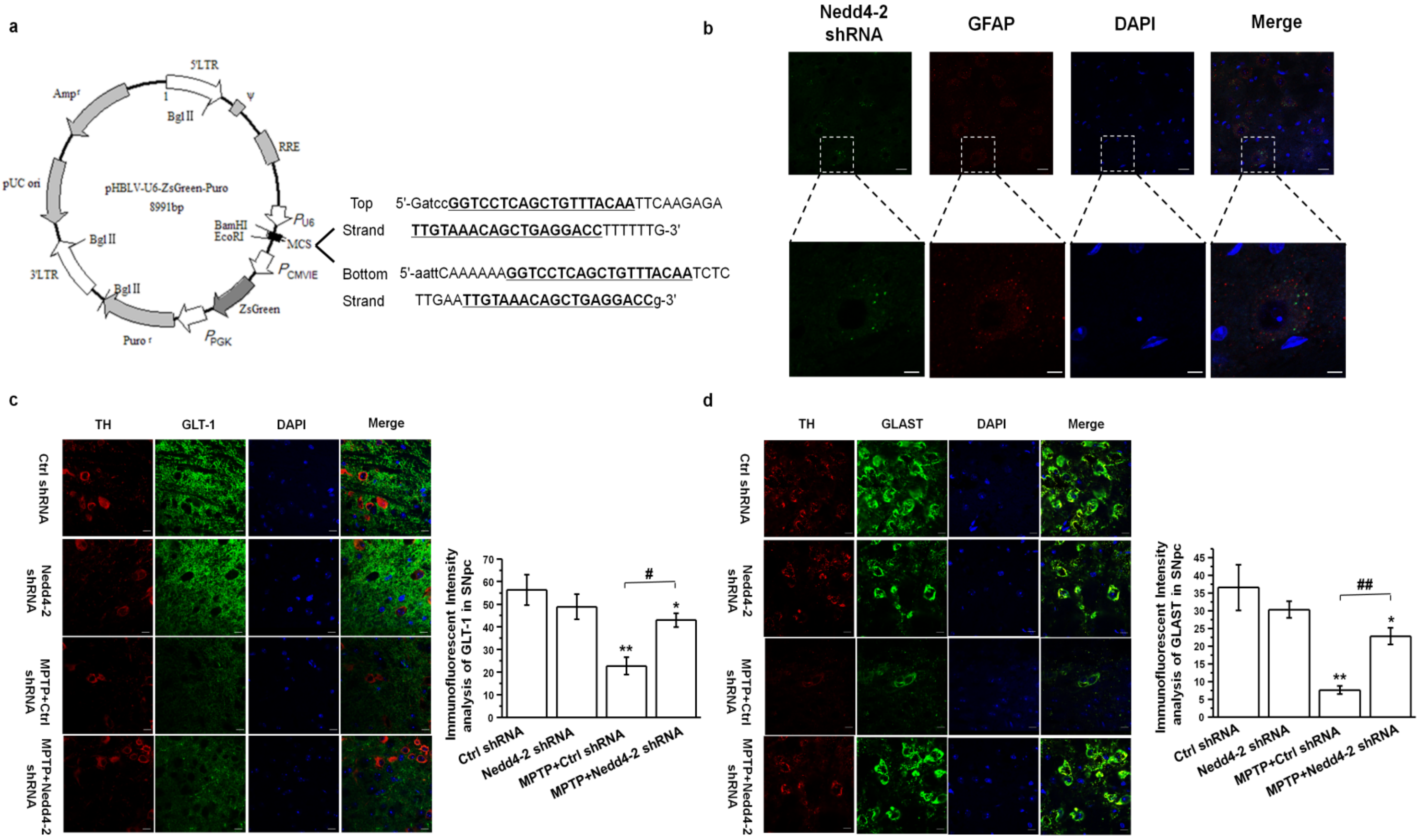

Supplementary Figure 8 (**a**) Design of an Nedd4-2 lentivirus vector (LV) (LV-sh[NEDD4-2]) with PGK-driven puromycin resistance and CMV IE-driven ZsGreen expression. LV-sh[NEDD4-2] was constructed to express shRNA targeting *Nedd4-2* from the U6 (RNA polymerase III) promoter and were generated by ligating annealed oligonucleotides encoding shNedd4-2 (in the figure) or a control sequence into the *Bam*H I/*Eco*R I sites. (**b**) Images of Nedd4-2 shRNA (GFP) co-labeling with GFAP confirms that the lentivirus vector targets astrocytes in the SN region. Scale bar: 40 μm at the top and 20 μm at the bottom . (**c** and **d**) Immunofluorescent images of TH co-labelling with GLT-1 or GLAST. Scale bar: 30 μm.

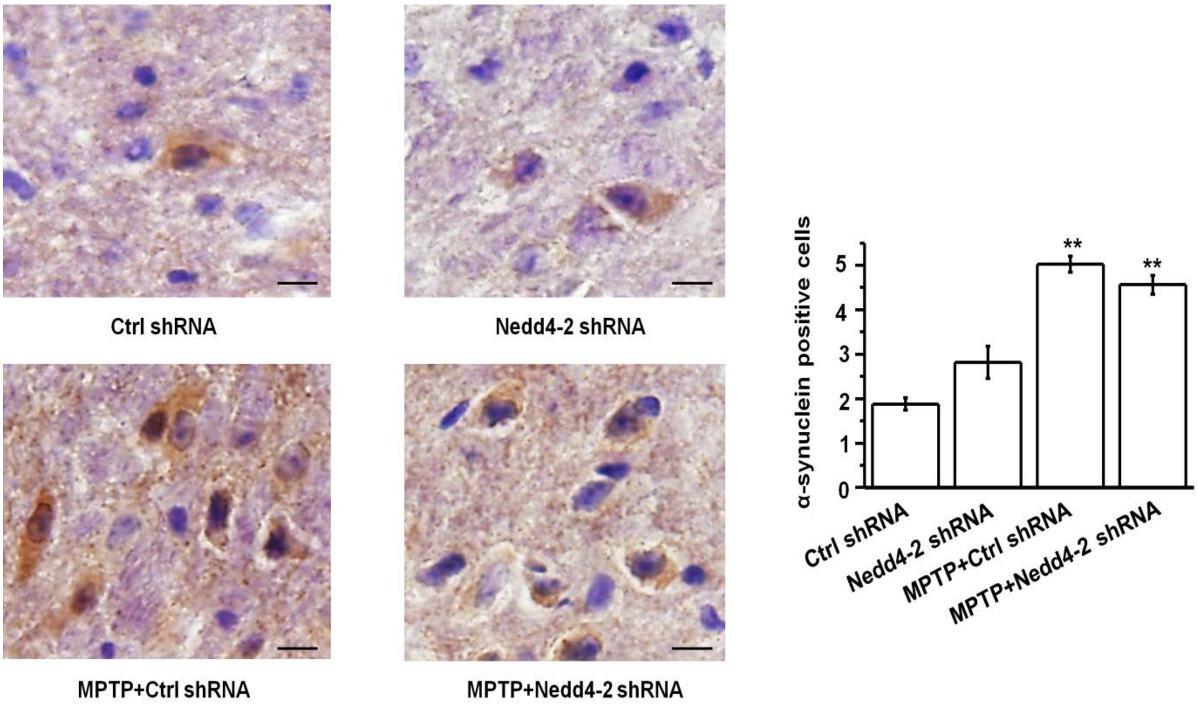

Supplementary Figure 9 Effect of Nedd4-2 knockdown on the α-synuclein expression in the SN. IHC staining showing that Nedd4-2 knockdown has no obvious effects on α-synuclein expression in the SN of MPTP-treated mice. Results are expressed as the mean ± SE. One-way ANOVA. n = 6 per group. $^{**}p < 0.01$. Scale bar: 40 μm.
